# The Rice Pentatricopeptide Repeat Gene *TCD10* is Needed for Chloroplast Development under Cold Stress

**DOI:** 10.1186/s12284-016-0134-1

**Published:** 2016-12-01

**Authors:** Lanlan Wu, Jun Wu, Yanxia Liu, Xiaodi Gong, Jianlong Xu, Dongzhi Lin, Yanjun Dong

**Affiliations:** 1 Development Center of Plant Germplasm Resources, College of Life and Environment Sciences, Shanghai Normal University, Shanghai, 200234 China; 2 Institute of Genetics and Developmental Biology, Chinese Academy of Sciences, No.1 West Beichen Road, Chaoyang District Beijing, 100101 China; 3 The Institute of Crop Sciences, Chinese Academy of Agricultural Sciences, 12 South Zhong-Guan Cun Street, Beijing, 100081 China

**Keywords:** Albino phenotype, Chloroplast development, Cold stress, PPR protein, Rice

## Abstract

**Background:**

Chloroplast plays a vital role in plant development and growth. The pentatricopeptide repeat (PPR) gene family is one of the largest gene families in plants. In addition, cold stress affects a broad spectrum of cellular components, e.g. chloroplast, and metabolism in plants. However, the regulatory mechanism for rice PPR genes on chloroplast development still remains elusive under cold stress.

**Result:**

In this paper, we characterized a new rice PPR gene mutant *tcd10 (thermo-sensitive chlorophyll-deficient mutant 10)* that exhibits the albino phenotype, malformed chloroplast and could not survive after the 5-leaf stage when grown at 20 °C, but does the normal phenotype at 32 °C. Map-based cloning, followed by RNA interference and CRISPR/Cas9 genome editing techniques, revealed that *TCD10* encoding a novel PPR protein, mainly localized to the chloroplasts, with 27 PPR motifs, is responsible for the mutant phenotype. In addition, *TCD10* is specific expression in tissues. The disruption of *TCD10* resulted in an evidently reduced expression of chloroplast-associated genes under cold stress (20 °C), whereas they did recovered to normal levels at high temperature (32 °C). These results showed an important role of *TCD10* for chloroplast development under cold stress.

**Conclusions:**

The *TCD10* encodes a novel rice PPR protein, mainly located in chloroplasts, which is important for chloroplast development, growth and the maintenance of photosynthetic electron transport and its disorder would lead to an aberrant chloroplast and abnormal expressions in these genes for chloroplast development and photosynthesis in rice under cold stress.

**Electronic supplementary material:**

The online version of this article (doi:10.1186/s12284-016-0134-1) contains supplementary material, which is available to authorized users.

## Background

The formation of normal chloroplasts is crucial for photosynthesis and carbon assimilation in plants. Chloroplast is a semi-autonomous organelle which has its own DNA genome and protein synthesis system, but the only part of the proteins can be synthesized in the chloroplast, the vast majority of proteins are synthesized on the ribosomes of the cytoplasm. Chloroplasts are mostly allocated in the leaves of plants, and its development is closely linked with the leaf development. As known, plastid development from proplastids to mature chloroplasts can be divided into three phases (Chory et al. 1991; Mullet 1993; Kusumi et al. 1997 and 2014). The first phase concerns the activation of plastid replication and plastid DNA synthesis. The second phase is the chloroplast “build-up” stage, which is characterized by the construct of the chloroplast genetic system. At this phase, the nuclear-encoded plastid RNA polymerase (NEP) preferentially transcribes the plastid genes and transcription/translation in the chloroplasts increase strikingly (Hajdukiewicz et al. 1997). In the final phase, the plastid- and nuclear genes for photosynthetic apparatus are massively expressed and principally transcribed by the plastid-encoded RNA polymerase (PEP) (De Santis-Maciossek et al. 1999). Plastid-encoded proteins assemble with imported nuclear-encoded proteins to form photosynthetic and metabolic complexes in chloroplasts. Thus, chloroplast development needs tightly coordinated gene expression between the chloroplast- and nuclear genomes.

Pentatricopeptide repeat (PPR) proteins are one of the largest protein families in plants. The PPR family is characterized by tandem arrays of the PPR motif, a highly degenerate repeat of 35 canonical amino acids (Small and Peeters 2000). PPR proteins harbor between two and 30 PPR motifs, and their tandem alignment allows the modular recognition of specific RNA sequences, generally function in chloroplast or mitochondria (Fujii et al. 2013). Recent studies showed that PPR proteins can promote versatile processes involving RNA, such as editing, splicing, stabilization, and translation (Schmitz-Linneweber and Small 2008; Fujii and Small 2011; Nakamura et al. 2012). In chloroplasts, some PPR proteins have been identified to participate in RNA splicing (Schmitz-Linneweber et al. 2006; De Longevialle et al. 2007; Ichinose et al. 2012), RNA processing (Meierhoff et al. 2003; Hattori et al. 2007), RNA editing (Kotera et al. 2005; Okuda et al. 2007; Chateigner-Boutin et al. 2008; Cai et al. 2009; Yu et al. 2009; Zhou et al. 2009; Tseng et al. 2010; Sosso et al. 2012), translation (Williams and Barkan 2003; Tavares-Carreón et al. 2008), and RNA stability (Yamazaki et al. 2004; Pfalz et al. 2009; Fujii et al. 2013). Despite the fact, a lot of work still to be done is to discriminate the functions of PPR genes in plant development, especially in rice.

Rice (*Oryza sativa*) is the most important food crop and has also been established as a model species for plant genome research. It is known that a mutation in a PPR gene usually has a strong phenotypic effect (Kocábek et al. 2005). Notably, functional studies of rice PPR proteins remain much sparse. OsPPR1, carrying 11 PPR motifs, is the first identified PPR protein responsible for rice chloroplast development (Gothandam et al. 2005). Antisense transgenic approach was exploited to suppress the expression of *OsPPR1* and the obtained transgenic lines displayed chlorophyll-deficiency, albinism and lethality. Another rice PPR protein, YSA, with 16 PPR motifs, also is essential for chloroplast development and its disruption causes a seedling stage-specific albino phenotype (Su et al. 2012). Also the chloroplast-localized PPR protein OsV4 is needed for chloroplast development at early seedling stage under cold stress (Gong et al. 2014). More recently, also we reported that rice ASL3 with 10 PPR motifs is required for chloroplast development and its disruption lead to the death of seedlings (Lin et al. 2015a, b). Herein, we describe a novel thermo-sensitive chlorophyll-deficient mutant, *tcd10*, which displayed the albino phenotype below 20 °C and *TCD10* encodes a novel PPR protein, containing 27 PPR motifs, required for chloroplast development and photosynthesis in rice under cold stress.

## Results

### Phenotypic Analysis of the *tcd10* Mutant

**Fig. 1:**
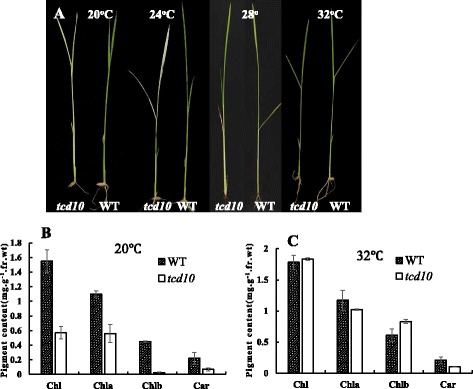
Characterization of the *tcd10* mutants; **a** 4-leaf stage seedlings of wild type (WT) (right) and *tcd10* mutants (left) grown at 20 °C, 24 °C, 28 °C and 32 °C, respectively; **b**, **c** photosynthetic pigment contents of *tcd10* and WT 3-leaf stage seedlings at 20 °C and 32 °C respectively; Chl, total chlorophyll; Chla, chlorophyll a; Chlb, chlorophyll b; Car, carotenoid

The color of the *tcd10* mutant seedlings grown at 20 °C, 24 °C, 28 °C and 32 °C, respectively, was shown in Fig. 1a. Apparently, the mutant seedlings displayed albino phenotype, thereafter could not survive past 5-leaf stage at 20 °C(data not shown); the degree of chlorosis gradually weakened when grown at 24 °C and 28 °C, but become green as WT plants at 32 °C, indicating the low thermo-sensitivity of the mutant phenotype. Consist with the observed phenotype, the accumulations of chlorophyll a, b, and carotenoid in *tcd10* plants at 20 °C (Fig. 1b) were drastically lower than those at 32 °C and WT plants (Fig. 1c). It is indicated that the low temperature led to the block of formation of photosynthetic pigments in *tcd10* mutants.

**Fig. 2:**
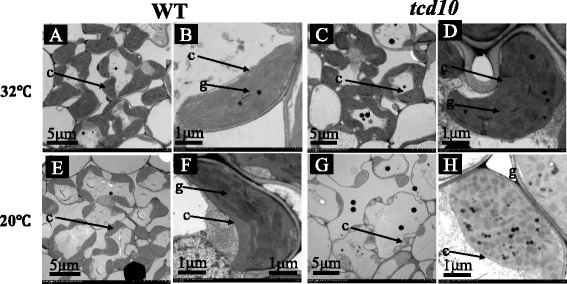
Transmission electron microscopic images of chloroplasts in WT and *tcd10* mutant; **a**, **b**, **c**, **d**, Chloroplast structure in WT(**a**, **b**) and tcd10 (**c**, **d**) cell at 32 °C; **e**, **f**, **g**, **h**, Chloroplast structure in WT(**e**, **f**)and tcd10(**g**, **h**) cell at 20 °C. c, chloroplast; **g**, grana lamella stacks

The alterations of leaf-color are generally accompanied with the development of chloroplast. To verify this view, we compared the chloroplast ultrastructure of *tcd10* and WT leaves under 32 °C and 20 °C. As expected, WT cells (Fig. 2) under 32 °C and 20 °C contained normal chloroplasts with well-organized lamellar structures and were equipped with normally stacked grana and thylakoid membranes. By contrast, most cells under 20 °C in *tcd10* mutants contained fewer chloroplasts (Fig. 2g), which were theteroplastidic and contained non-pigmented plastids with severely vacuolated and lacked organized lamellar (Fig. 2h). However, the mutant cells at 32 °C had not obvious difference with WT plants (Fig. 2c, d). Accordingly, the *tcd10* aberrant chloroplast induced by low temperatures caused the decreased accumulation of photosynthetic pigments, so as to produce the mutant phenotype.

We investigated the *Fv/Fm* values, reflecting the maximum potential capacity of the PSII photochemical reactions in chloroplasts (Krause and Weis 1991). Resultantly, at 20 °C, the *Fv/Fm* value was 0.834 ± 0.04 in WT plants, and undetectable in *tcd10* mutants, indicating that the photochemical efficiency of PSII was completely hindered in *tcd10* mutants under cold stress. In addition, the max rate (molm^−2^s^−1^) of photosynthetic electron transport (*rETR*_*max*_) was 63.34 ± 0.484 in WT plants and undetectable in *tcd10* mutants, showing that the *tcd10* mutation completely blocked the electron transport under cold stress. By contrast, at 32 °C, all values in the *tcd10* mutants (0.559 ± 0.012 for *Fv/Fm* and 157.200 ± 1.131 for *rETR*_*max*_, respectively) were nearly similar to WT levels (0.642 ± 0.024 for *Fv/Fm* and 143.800 ± 8.707 for *rETR*_*max*_, respectively), showing that both PSII activity and photosynthetic electron transport in the *tcd10* mutants were nearly recovered to WT levels at 32 °C.

In addition, the leaf chlorophyll SPAD values (Additional file 1: Figure S1) in *tcd10* plants had not significant difference from WT plants after transplanting under nature conditions. However, in contrast to have not great influence on both the final plant height (Additional file 2: Figure S2) and 1000-grain weight (Additional file 3: Figure S3), the panicle number and panicle length was significantly reduced (Additional file 3: Figure S3). These results illustrated the *tcd10* mutation affects not only chloroplast development under cold stress and but also the late growth of rice under nature conditions.

### Map-based Cloning of *TCD10*

**Fig. 3:**
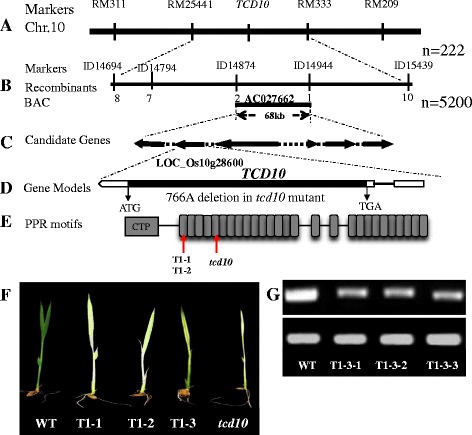
Genetic analysis and cloning of the *TCD10* gene; **a** *TCD10* was located between the RM25441 and RM333 on chromosome 10 using 222 F2 mutant individuals; **b** *TCD10* was narrowed to a 70 kb between ID14874 and ID14944 on AC027662 BAC clone using 5200 F2 mutant individuals; **c** The target region contains six predicted candidate genes (*LOC_Os10g28590, LOC_Os10g28600 LOC_Os10g28610, LOC_Os10g28620, LOC_Os10g28630, LOC_Os10g28640*); **d** A single nucleotide (A) deletion mutation at position 766 bp from the ATG start codon in *LOC_Os10g28600*; **e** Chloroplast transit peptide (CTP), PPR motif and mutation sites; **f** Phenotypes of CRISPR/Cas9 genome editing (T1-1 and T1-2) and RNAi (T1-3) transgenic lines grown at 20 °C; **g** Transcript levels of three RNAi (T1-3) transgenic lines

**Fig. 4:**
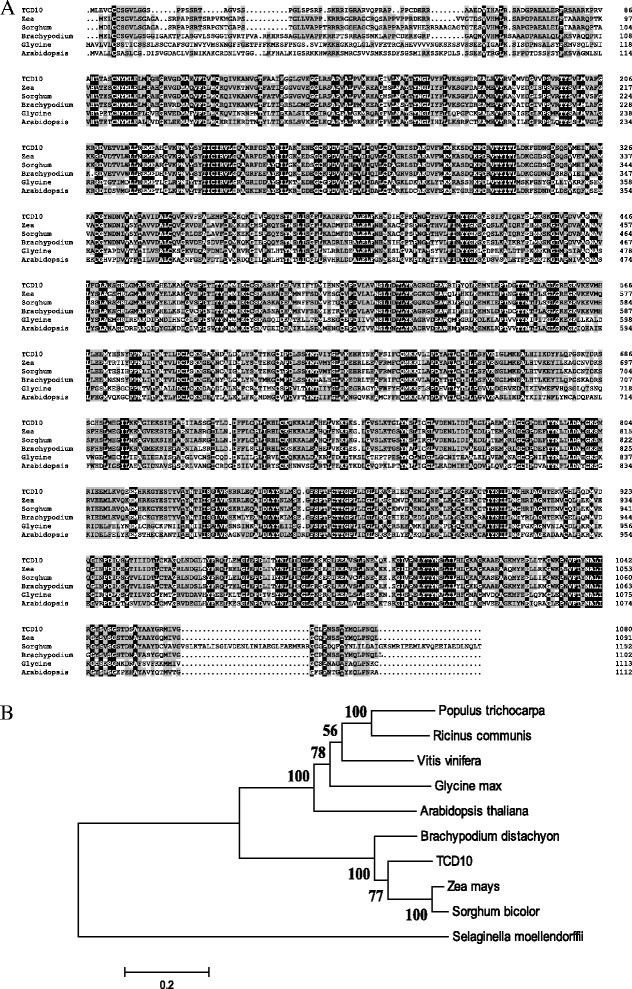
Phylogenic analysis of TCD10; **a** Amino acid sequence alignment of TCD10 with the five homologs. Amino acids fully or partially conserved are shaded black and gray, respectively. The homologous comparison is based on a multiple sequence with alignment algorithm MAFFT and generated with the programDNAMAN8; **b** Phylogenic tree of TCD10 and homologs. Protein sequences are *Ricinus communis* (RCOM_ 8274176), *Populus trichocarpa* (POPTR_0006s28060), *Arabidopsis thaliana* (AT4G31850), *Glycine max* (100778358), *Vitis vinifera* (100247529), *Brachypodium distachyon* (100836501), *Sorghum bicolor* (SORBI_01g032160), *Zea mays* (GRMZM2G372632), *Selaginella moellendorffii* (SELMODRAFT_76934). Scale represents percentage substitution per site. The rooted tree is based on a multiple sequence with alignment algorithm MAFFT and generated with the program Mega6

The genetic analysis (Additional file 4: Table S1) showed that this mutation in *tcd10* is a single recessive locus based on the approximate 3(green):1(albino) ratio for phenotypic segregation using F_2_ population generated from Guangzhan63S*/tcd10*. To this end, we randomly selected the 222 F_2_ individuals with mutant phenotype and mapped *TCD10* between RM475 and RM33 on chromosome 10 (Fig. 3a). To further fine-map *TCD10*, a total of 5200 F_2_ mutant invididuals and developed five InDel molecular markers (Additional file 5: Table S2) were exploited. Ultimately, the *TCD10* was localized to 70 kb between ID14874 and ID14944 (Fig. 3b). Using Rice Genome Annotation Project (http://rice.plantbiology.msu.edu/cgi-bin/gbrowse/rice/#search), the target genomic region contained six predicted candidate genes (Fig. 3c). By sequencing and analyzing all the candidate genes, compared with wild type, in the *tcd10* mutants we only uncovered a 1-bp deletion (A) at position 766 bp from the ATG start codon in the extron of *LOC_Os10g28600* (Fig. 4d) which caused pre-termination, suggesting that *TCD10* is *LOC_Os10g28600*.

### Knock-down and -out of *TCD10* Exhibits the Mutant Phenotype

To further affirm that the malfunction of *TCD10* is blame for the mutant phenotype, we used both RNA interference (RNAi) and CRISPR/Cas9 genome editing system to knockdown and knockout *LOC_Os10g28600* in WT plants, respectively. In RNAi study, we obtained eleven-one RNAi lines all showing the chlorosis phenotype similar to *tcd10* at 20 °C(Fig. 3f). Thus, the down-regulated *TCD10* expression in RNAi lines (Fig. 3g) could mimic the phenotype of the *tcd10* mutant. In CRISPR/Cas9 study, we obtained 38 transgenic T_0_ plants carrying two different editing sites (the 1-bp (G) and 17-bp (CCATGGCCGGGTCGGGG) deletion happened at position 316 bp and 306 bp from the ATG start codon in *TCD10*, respectively) (Additional file 6: Figure S4). Importantly, all transgenic T_1_ plants appeared the separation of albino phenotype as *tcd10* mutants at 20 °C (Fig. 3f). Taken together, these results reconfirmed that *LOC_Os10g28600* is *TCD10.*

### Characterization of TCD10 Protein

Determination of the nucleotide sequence of the RT-PCR product revealed that *TCD10* encodes a 3790 bp open-reading frame, which is not interrupted by any intron and encodes a 1080-amino acid polypeptide with a calculated molecular mass of 120kD. The TCD10 contains 27 PPR motifs of the protein (Fig. 3e, Additional file 7: Figure S5). In addition, the first 50 amino acids (Additional file 7: Figure S5) were predicted to be a chloroplast transit peptide (CTP) by targetp (http://www.cbs.dtu.dk/services/TargetP/). In addition, the two editing deletion mutations in the transgenic lines (T1-1and T1-2) and the *tcd110* mutation occurred in the 1st and 5th PPR motif, respectively, and all caused the premature termination of translation, finally leading to the complete damage of protein structure (Fig. 3e, Additional file 7: Figure S5). Multiple amino acid sequence alignment showed that TCD10 was similar to many species (Fig. 4a), including Arabidopsis At4g31850/PGR3 with 62.3% of identity (Shikanai et al. 1999; Yamazaki et al. 2004; Cai et al. 2011; Fujii et al. 2013). The subsequent phylogenetic analysis showed that TCD10 had the closest genetic relationship with one of the predicted proteins in sorghum (Fig. 4b), indicating the high conservation of TCD10 protein in plants.

### *TCD10* Expression Pattern

**Fig. 5:**
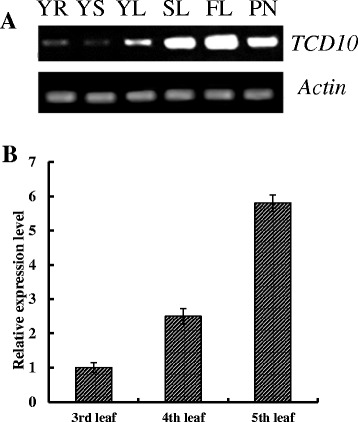
Expression patterns of *TCD10* by RT-PCR analysis; **a** Expression of various tissues; YR, young-seedling roots; YS, young-seedling stem; YL, young-seedling leaf; SL, second leaf; FL, flag leaf at heading; PN, young panicles. *OsActin* was used as a control (cycle number for *OsActin* was 28, cycle number for *TCD10* was 35); **b** Expression levels at different leaves

To determine expression pattern of *TCD10* gene in various tissues, we analyzed the *TCD10* expression level of the root, stems, young-leaf, flag-leaf and panicle in wild-type plant tissues. As shown in Fig. 5a, *TCD10* in all various tissues were expressed, but in the leaves was relatively higher than that of other tissues, especially in the flag leaf expression was the highest, and expression in roots and stems rarely, which basically is consistent with RiceXPro database prediction results (http://ricexpro.dna.affrc.go.jp/) (Additional file 8: Figure S6). In addition, the expression levels were increased together with leaf age (Fig. 5b), indicating the importance of *TCD10* for leaf chloroplast development under cold stress.

### The Transcript Expressions of the Associated Genes in the *tcd10* Mutants

**Fig. 6:**
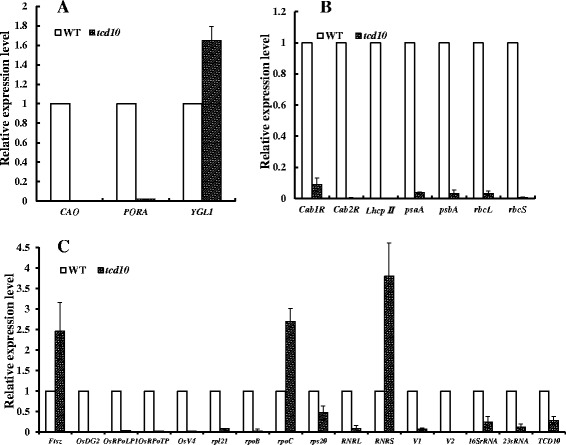
Transcript levels of those genes associated with Chl biosynthesis (**a**), photosynthesis (**b**) and chloroplast development (**c**) at 20 °C in WT and *tcd10* mutants in the 3rd leaves, respectively. The relative expression level of each gene in WT and mutant was analyzed by qPCR and normalized using the *OsActin* as an internal control. Data are means ± SD (*n* = 3)

**Fig. 7:**
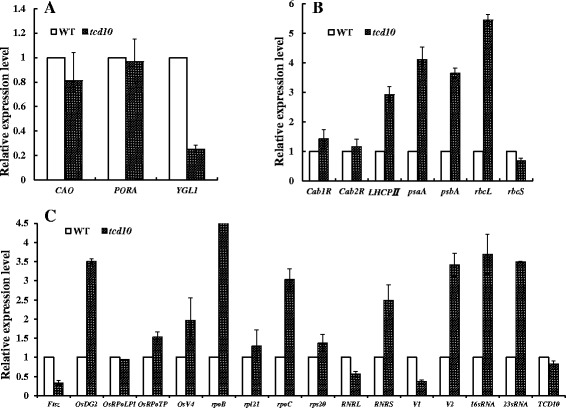
qPCR analysis of those genes associated with Chl biosynthesis, photosynthesis and chloroplast development at 32 °C in the 3rd leaves; **a**, **b**, **c** Expression levels of genes related to Chl biosynthesis (**a**), photosynthesis (**b**) and chloroplast development (**c**) in WT and *tcd10* mutants in the 3rd leaves, respectively

We investigated the transcript levels of twenty-five genes (Additional file 9: Table S3) associated with Chl biosynthesis, photosynthesis and chloroplast development (*CAO1*, *PORA, YGL1, Cab1R, Cab2R, rbcL, rbcS, psaA, psbA, LhcpII, rps7, OsV4, rpoC, OsRpoTp, V1, V2, RNRL, RNRS, 16SrRNA, rps20, FtsZ, rpl21,* and *OsDG2*) in the *tcd10* mutants. As a result, under cold stress (20 °C) (Fig. 6a), except for the slight increase in *YGL1* encoding Chl synthase, compared with WT plants, the transcripts in those genes for Chl biosynthesis, such as chlorophyllide a oxygenase (*CAO1*), and NADPH: protochlorophyllide oxidoreductase (*PORA*), were significantly impeded in *tcd10* mutants, consistent with the reduced Chl content (Fig. 1c) and the mutant phenotype (Fig. 1a). Also all the seven photosynthesis-associated genes studied (*Cab1R, Cab2R, rbcL, rbcS, psaA, psbA, LhcpII*) (Fig. 6b) were severely hampered in *tcd10* mutants, consistent with the undetectable photochemical efficiency of PSII and photosynthetic electron transport in photosystems in *tcd10* mutants, thereafter led to the seedling death. Moreover, all transcript accumulations of sixteen genes tested for chloroplast development were abnormally influenced in *tcd10* mutants, especially *OsV4, OsRpoTp, V1, V2, RNRL, RNRS, 16SrRNA, rpl21* and *OsDG2* were considerably down-regulated (Fig. 6c). More notably, at 32 °C, the transcript levels of the down-regulated genes at low temperatures in *tcd10* mutants were recovered to or even slightly higher than WT plants (Fig. 7a, b, c). Accordingly, under high temperatures (32 °C), the *tcd10* mutation has very little effect on the associated-genes for Chl biosynthesis, photosynthesis and chloroplast development, consistent with the normal phenotype in *tcd10* mutants at 32 °C. Taken together, *TCD10* is needed for chloroplast development under cold stress.

### Subcellular Localization of TCD10

**Fig. 8:**
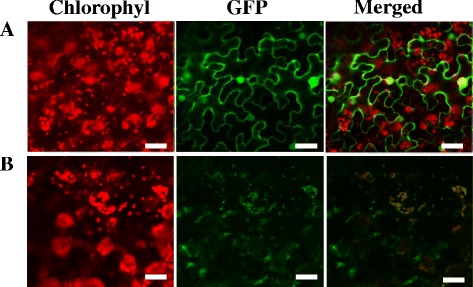
Subcellular localization of TCD10 protein; **a** Empty GFP vector without a specific targeting sequence; **b** TCD10-GFP fusion; The scale bar represents 20 μm

The majority of PPR proteins are predicted to be targeted to either mitochondria or chloroplasts (Small and Peeters 2000). In this study, TargetP (Emanuelsson et al. 2000, http://www.cbs.dtu.dk/services/TargetP/) and iPSORT (http://ipsort.hgc.jp/) all suggested possible chloroplast localization and secretory channels. To assess possible localization of TCD10, we constructed the recombinant expression vectors pMON530-GFP-TCD10 and transformed into tobacco cells by Agrobacterium-mediated infection. Meanwhile, empty GFP vector was used as a control. Resultantly, the vast majority of the green fluorescent signal of the TCD10-GFP fusion protein was coincident with the chlorophyll autofluorescence, and the remaining little parts did not overlap in tobacco cells (Fig. 8b). In contrast, the cells transformed with the empty GFP vector without a specific targeting sequence had green fluorescent signals in both the cytoplasm and the nucleus (Fig. 8a). These findings clearly suggest that TCD10 was mainly localized to the chloroplast in addition to secretory channels.

## Discussion

### *TCD10* is Needed for Chloroplast Development and Growth under Cold Stress

The chloroplast is a semi-autonomous organelle, which contains about 100 genes, more than 3000 proteins function within it (Leister 2003). The normal chloroplast development needs tightly coordinated expressions between plastid- and nuclear genes. The mutation of those genes could result in chlorophyll deficient/chloroplast defects in plants. In this study, the *tcd10* mutants, under low temperatures (20 °C), had the severe affected transcript-levels of most genes for Chl biosynthesis, photosynthesis and chloroplast development (Fig. 6), whereas the down-regulated genes at low temperatures could be recovered to normal level or even higher WT levels at high temperature (32 °C) (Fig. 7). This discrepancy was attributable to the difference in the chloroplast structure and pigment contents between 20 °C and 32 °C. In addition, as known, when rice seedlings grow up to the 3/4-leaf stage, a seed’s energy is exhausted, stored reserves become insufficient to meet itself the rice growth demand. The continued growing of seedlings requires the external supply of resources from photosynthesis. Hence, we could speculate that the no survival for *tcd10* mutants after the 5-leaf stage at 20 °C maybe result from the loss of photosynthesis due to the block of electron transport in photosystems under cold stress. Notwithstanding the reason why abnormal chloroplast occurs only under cold stress is not completely claimed yet, it is speculated that the *TCD10* function is possibly not prerequisite at higher temperatures, but it is essential/more required for rice chloroplast development under cold stress.

### *TCD10* Functions at the First Phase of Chloroplast Development Possible via Plastid Division

As described previously, the processes accompanying chloroplast development can be divided into three phases (Mullet 1993; Kusumi et al. 2010 and 2014). It is clearly known that *OsPOLP1*, encoding one plastidial DNA polymerase (Takeuchi et al. 2007; Kusumi et al. 2010), *FtsZ* encoding a component of the plastid division machinery (Vitha et al. 2001) are critical for the first phase, determine the number and size of chloroplasts (Takeuchi et al. 2007) and the NEP-transcribed genes which encode PEP cores (e.g. *rpoA, rpoB, rpoC*) for chloroplast genetic system and the PEP-transcribed genes (e.g. *psab, LhcpII*) involving PSII function at the second and third phase, respectively. Our data that all transcripts for the PEP-transcribed plastid (e.g. *rbcL*, *psaA* and *LhcpII*) and nuclear genes (*rbcS*) associated with photosynthesis apparatus (Fig. 6b) were obviously down-regulated in the *tcd10* mutants showed that *TCD10,* at least, regulates the third phase of chloroplast development. In addition to *V1* for NUS1 protein (Kusumi et al. 1997 and 2011), *V2* for guanylate kinase (Sugimoto et al. 2007) and *OsV4* for PPR protein (Gong et al. 2014) and *ASL2* encoding plastid ribosomal protein L21 (Lin et al. 2015a, b) and *TCM5* for Deg protease protein (Zheng et al. 2016), the extremely decreased accumulation of *OsRpoTp* (Hiratsuka et al. 1989) and the NEP-transcribed gene *rpoB* and *rpoC* which encodes PEP core β, β’ subunits functioning in the second step suggests that *TCD10* also affects the second phase of chloroplast development. Similarly, the significantly reduced transcripts of *rbcL*, an indicator for the accumulation of polysomes (Barkan 1993), chloroplast *16SrRNA, 23SrRNA* and *rps7, rps14, rps20* in the *tcd10* mutants indicated that the *tcd10* mutation affects the assembly and accumulation of plastid ribosomes, resulting in disruption of plastid translation. Similarly, the blockage of the chloroplast *16SrRNA* maturation in Arabidopsis *rap* mutant (Kleinknecht et al. 2014) and rice *al1* mutant (Zhang et al. 2016) leads to abnormal chloroplasts. Thus, *TCD10* participates in not only the plastid translation, but also the assembly and the accumulation of plastid ribosomes. More notably, the obviously down-regulations in transcripts of the known key genes (*OsPoLP1* for one plastidial DNApolymerase (Takeuchi et al. 2007) (>25 fold) and *RNRL* for (Yoo et al. 2009) (>11 fold), *OsDG2* for glycine-rich protein (Jiang et al. 2014a) (>166 fold) at the first step of chloroplast development indicated that *TCD10* functions in the first step. As for the slightly increase in *FtsZ* transcript (<3 fold), it is likely due to the feedback effects. In addition, in view of fewer and smaller of chloroplasts (Fig. 2g) and the abnormal thylakoid structure (Fig. 2h) in *tcd10* mutant cells, it suggests that the *TCD10* regulates the first phase of chloroplast development probably via plasmid division under cold stress.

### Possible Role of TCD10 Protein in Chloroplast RNA Metabolism

PPR protein can specifically identify single stranded RNA sequence, and it is found that the PPR protein is closely related to the transcription process, the shearing process, the editing process and the stability of RNA (Kotera et al. 2005; Okuda et al. 2007; Chateigner-Boutin et al. 2008; Cai et al. 2009; Yu et al. 2009; Zhou et al. 2009; Tseng et al. 2010; Sosso et al. 2012; Williams and Barkan 2003; Tavares-Carreón et al. 2008; Yamazaki et al. 2004; Pfalz et al. 2009; Fujii et al. 2013). With the unveiling of the primary version of the PPR code, manipulation of PPRs to bind to target RNAs is within reach (Barkan et al. 2012), biochemical (Barkan et al. 2012; Kobayashi et al. 2012), statistical (Barkan et al. 2012; Yagi et al. 2013), and evolutionary constraint (Fujii and Small 2011) studies have postulated that the 1st, 3rd, and 6th amino acids within the PPR consensus manipulates RNA base recognition specificity. As stated above, at low temperatures, *TCD10* directly or indirectly regulates RNA transcript levels at all three steps of chloroplast development and participates in the plastid translation, the assembly and the accumulation of plastid ribosomes. As for *TCD10*, how to specifically participate in chloroplast RNA metabolism is unclear yet in detail. However, interestingly, Arabidopsis PGR3 (At4g31850), containing the same 27 PPR motifs, with the highest homolog (62.3% of identity) to TCD10 (Fig. 4a and Additional file 7: Figure S5), was previously studied in very detail (Shikanai et al. 1999; Yamazaki et al. 2004; Cai et al. 2011; Fujii et al. 2013) by mean of three Arabidopsis *pgr3-1*(the substitution mutation site in the 15th PPR motif), *pgr3-2*(the substitution mutation in the 12th PPR motif)*,* and *pgr3-3* (the substitution mutation in the 27th PPR motif) mutant alleles (Additional file 7: Figure S5). The chloroplast-targeted PGR3 was reported to have three distinct functions: (i) stabilization of photosynthetic electron transport L (petL) operon RNA, (ii) translational activation of *petL*, and (iii) translational activation of *ndhA* (Yamazaki et al. 2004; Cai et al. 2011) through binding to the 5′untranslated region (UTR) of petL operon RNA and *ndhA* (Cai et al. 2011). More interestingly, the 16 N-terminal PPRs in PRG3 was sufficient for function (i) via sequence-specific RNA binding, whereas the 11 C-terminal motifs were essential for functions (ii) and (iii) by activating translation (Fujii et al. 2013). Considering the high homolog between TCD10 and PGR3, it could be speculated that TCD10 may have similar three functions. Since the mutation in *tcd10* mutants occurred in the 5th PPR motif site (Fig. 3e), resulting in the changes of structure and loss-of-function of TCD10, thus the *tcd10* mutants should loss all three functions aforementioned. Indeed, the severe defects in photosynthetic electron transport pathways in *tcd10* mutants as *pgr3* mutants (Yamazaki et al. 2004) strongly supported the notion that *TCD10* has the stabilization (i) of photosynthetic electron transport under cold stress; Meanwhile, the reduced transcripts of protochlorophyllide oxidoreductase also indirectly indicates the block of (iii) translational activation of *ndhA.* Regrettably, no evidences in our study can support the (ii) function of translational activation of *petL* because of our technical limitations. It is notable that, only under cold stress, *TCD10* affected chloroplast development unlike Arabidopsis *PRG3* without temperatures, showing their obviously different responses to temperatures. These findings could highlight the notion that highly conserved genes within various species might play diverse and complex roles than previously recognized. Hence, further understanding function difference between PGR3 and TCD10 and how *TCD10* functions chloroplast development under cold stress is needed.

## Conclusions

The rice *TCD10* encodes a new PPR protein, mainly located in chloroplast, which is important for chloroplast development, seedling growth and the maintenance of photosynthetic electron transport and its disorder would lead to an aberrant chloroplast and abnormal transcript levels of genes associated with chloroplast development and photosynthesis under cold stress.

## Methods

### Plant Materials and Growth Conditions

The thermo-sensitive chlorophyll-deficient mutant, *tcd10*, used in this study, was isolated from *japonica* rice variety, Xiushui11 (WT), treated by ^60^Co gamma-radiation in 2006. All rice plants were grown in a rice paddy field in Shanghai (31°11’N, summer season, temperate climate) and Hainan (18°16’N, winter season, subtropical climate), China under local conditions or in the growth chambers.

### Phenotype Observation and Photosynthetic Pigment Measurements

The germinated rice-seeds were grown at the chambers with four temperatures (20 °C, 24 °C, 28 °C and 32 °C) and 12-h-dark/12-h-light. Each third-leaf 200 mg were sampled at the 3-leaf stage and used to extract the pigments. Photosynthetic pigment (chlorophyll a, b carotenoid) were measured according to previously described methods (Jiang et al. 2014b).

To examine the changes of plant height and leaf chlorophyll content during the whole growth-period, leaf chlorophyll SPAD values, using fast non-destructive chlorophyll meter (SPAD-502, Minolta, Japan) (Peng et al. 1993; Turner and Jund, 1991), and plant height were measured once a week from the 1st weeks after transplanting, respectively. At maturity, panicle-associated traits of rice plants (Additional file 1: Figure S1) were investigated.

### Chlorophyll Fluorescence and Transmission Electron Microscopy Analysis

Rice seedlings were cultivated at 20 °C and 32 °C in growth chambers. Chlorophyll fluorescence analysis for the 3rd leaves at the 3-leaf stage was performed by mean of a PAM-2000 portable chlorophyll fluorometer (MINI-PAM; Walz; http://walz.com). In addition to the max rate of photosynthetic electron transport (*rETRmax*), the variables *Fo*, *Fm*, *Fv*, and the *Fv/Fm* ratio were measured and calculated basically according to Meurer et al. (1996) after dark-adapted for 20 min. In addition, the observation of chloroplast ultrastructure in the third leaves was performed according to the previously described methods (Jiang et al. 2014a, b). Finally, samples were examined with a Hitachi-7650 transmission electron microscope.

### Map-based Cloning of *TCD10*

Rice genomic DNA was extracted from young leaves by the CTAB method with minor modifications (Stewart and Via 1993). For genetic analysis and mapping for *TCD10* locus, we crossed *tcd10* mutant with Guangzhan63S (*indica*) to obtain F_2_ mapping population. Initially, we adopted 81 SSR primers based on data in Gramene (http://www.gramene.org) to determine the chromosome location of *TCD10*. Then new SSR and InDel markers (Additional file 5: Table S2) were designed using the PREMIRE 5.0 software based on the whole genomic sequences of the *japonica* Nipponbare variety (Goff et al. 2002) and the *indica* variety 9311 (Yu et al. 2002). In this study, a total of 5200 F_2_ mutant individuals were selected for fine-mapping. The functions of candidate genes and respective cDNA sequences were obtaining using TIGR (http://rice.plantbiology.msu.edu/cgi-bin/gbrowse/rice/) and KOME (http://cdna01.dna.affrc.go.jp/cDNA/index.html), respectively.

### RNAi Suppression and Knockout of *TCD10*

To confirm that *TCD10* was blame for the mutant phenotype observed, both RNAi and CRISPR/Cas9 genome editing technology, which is of significance for basic plant research and crop genetic improvement (Belhaj et al. 2015; Ma and Liu 2016; Ma et al. 2016), were preformed. In RNAi experiments, the construct vector pTCK303 with a maize ubiquitin promoter and a rice intron was used as an RNAi vector (Wang et al. 2004). Both anti-sense and sense versions of a specific 780 bp fragment from the coding region of the TCD10 were amplified, and successively inserted into pTCK303, to form the RNAi construct vector pTCK303-dsRNAiTCD10. The primer pairs are 5'CGAGCTCGGTTCCTGATGTTCTTGCCGTG3' (*Sac*I), 5' GACTAGTC GATCAGGTATGCATCCTTTCG 3' (*Spe*I) and 5' CGGGATCCCG GTTCCTGATGTTCTTGCCGTG 3 '(*BamH*I), 5' GGGGTACCCC GATCAGGTATGCATCCTTTCG 3' (*Kpn*I).

In CRISPR/Cas9 system experiments, to generate the Cas9 targeting construct for TCD10, using the CRISPR Primer Deigner software (Naito et al. 2015; http://crispr.dbcls.jp/), the annealed gRNA oligonucleotide pair (F: 5'GCCGCGCGGCCATGGCCGGGTCG3'; R: 5'AAAC CGACCCGGCCATGGCCGCG3') with recognition sequence was designed. Then, the recognition sequence was inserted into the region between the OsU6 promoter and the gRNA scaffolds, from pYLgRNA-OsU6 vector, of Cas9 expression backbone vector (pYLCRISPR/Cas9-MH) at the *Bsa*I sites according to the previously described method (Ma et al. 2015).

The resultant plasmid (pTCK303-dsRNAiTCD10 and CRISPR/Cas9 expression) and the respective empty vectors were introduced into Agrobacterium tumefaciens EHA105 and used to infect calli of WT plants according to the previously published methods (Hiei et al. 1994). The obtained T_0_ transgenic seedlings were grown in a paddy rice field after screening of tolerant-hygromycin and DNA sequencing according to our described methods (Jiang et al. 2014a, b). Then all T_1_ seedlings grown at 20 °C were used for surveying the segregation of phenotype.

### RNA Transcript Analysis

Total RNA was extracted from various fresh tissues by the TRIzol Reagent (Invitrogen; http://www.invitrogen.com) and DNase I treated by an RNeasy kit (Qiagen; http://www.qiagen.com) and the first-strand cDNA was synthesized with the Revert-Aid first-strand cDNA synthesis kit (Toyobo; http://www.toyobo.co.jp) following the manufacturer’s instructions. The specific primers used for RT-PCR assays were shown in Additional file 9: Table S3. Then semiquantitative RT-PCR analysis was performed to assay the tissue expression pattern of *TCD10*. Real Time qPCR (ABI7500, Applied Biosystems; http://www.appliedbiosystems.com) was used to analyze RNA expression levels for associated-genes of chloroplast development, chlorophyll synthesis and photosynthesis. The relative quantification of gene expression data was analyzed as described by Livak and Schmittgen (2001). Three independent experiments were performed, and each sample was run in triplicate. *OsActin* was used as an internal control.

### TCD10 Subcellular Localization

To investigate the subcellular localization of TCD10, cDNA fragments encoding the N-terminal region of 260 amino acids in TCD10 were amplified by PCR using following primer (5'-GAAGATCTATGTTGGAGGTCTGCTGCTGC-3' and 5'-GGGGTACCCCCTTCATTCTCCATTTTCGC-3') and introduced into vector pMON530-GFP at the *Bgl*II and *Kpn*I sites (the sequence underlined were represented cleavage sites). TCD10 localization was investigated by transient expression of GFP fusion in tobacco (*Nicotiana tabacum*) cells, and confocal microscopy as described previously (Jiang et al. 2014a, b). Finally, GFP fluorescence in the transformed protoplasts was imaged using a confocal laser-scanning microscopy (LSM 5 PASCAL; ZEISS, http://www.zeiss.com)

### Sequence and Phylogenetic Analysis

Gene prediction and structure analysis were performed using the GRAMENE database (www.gramene.org/). Homologous sequences of TCD10 were identified using the Blastp search program of the National Center for Biotechnology Information (NCBI, www.ncbi.nlm.nih.gov/). A phylogenetic tree of TCD10 and related proteins was then constructed by MEGA4 tool using the Neighbor-joining method with a bootstrap value of 1000 (Edgar 2004). The signal peptide was predicted with SignalP version 2.0 (Nielsen and Krogh 1998).

## Additional files

Additional file 1:
**Figure S1.** Changes of leaf chlorophyll SPAD from transplanting to maturity. (2010, Shanghai, China). (DOC 43 kb)
Additional file 2:
**Figure S2.** Changes of plant height from transplanting to maturity. (2010, Shanghai, China). (DOC 47 kb)
Additional file 3:
**Figure S3.** Comparison of panicle-related traits between *tcd10* mutant and wild type (2010, Shanghai, China); PN, Panicle number; PL, Panicle length (cm); GW, 1000-grain weight (g). (DOC 38 kb)
Additional file 4:
**Table S1.** Genetic segregation of *tcd10* mutants in the F_2_ population. (DOC 30 kb)
Additional file 5:
**Table S2.** The PCR-based molecular markers designed for fine mapping. (DOCX 14 kb)
Additional file 6:
**Figure S4.** Full-length cDNA sequence of *TCD10* and mutation sites; The nucleotides with the blue (A), red (G) and green (CCATGGCCGGGTCGGGG) letters represent the deleted nucleotides in *tcd10*, T1-1 and T1-2 transgenic lines using CRISPR/Cas9 system technique; The sequences in the box represents the recognition sequences in CRIPPER/Cas9 experiments. (DOCX 21 kb)
Additional file 7:
**Figure S5.** The protein sequences of TCD10 and PGR3 (At4g31850); The sequences with green letters indicate chloroplast transit peptide (CTP); The numbers represent PPR motifs; In TCD10, the amino acid with red letter indicate the deletion mutation sites in *tcd10* (the 1^st^ PPR) and T1-1, T1-2 transgenic lines (the 5^th^ PPR), respectively; In PGR3, the amino acid with red letter indicate the substitute mutation sites in *pgr3-1*(the 15th PPR), *pgr3-2*(the 12th PPR) and *pgr3-3*(the 27^th^ PPR) mutants. (DOCX 21 kb)
Additional file 8:
**Figure S6.** Expression patterns of *TCD10* (*LOC_Os10g28600)*. Data were obtained from the rice expression profile database, RiceXPro (http://ricexpro.dna.affrc.go.jp/category-select.php). (DOC 98 kb)
Additional file 9:
**Table S3.** Markers designed for realtime RT-PCR and gene functions. (DOCX 18 kb)
