# Supplementary figures and images for: The Rice Pentatricopeptide Repeat Gene TCD10 is Needed for Chloroplast Development under Cold Stress
Source: Rice (N Y). 2016 Dec 1;9:67. doi: 10.1186/s12284-016-0134-1 (PMC5133210; doi:10.1186/s12284-016-0134-1)

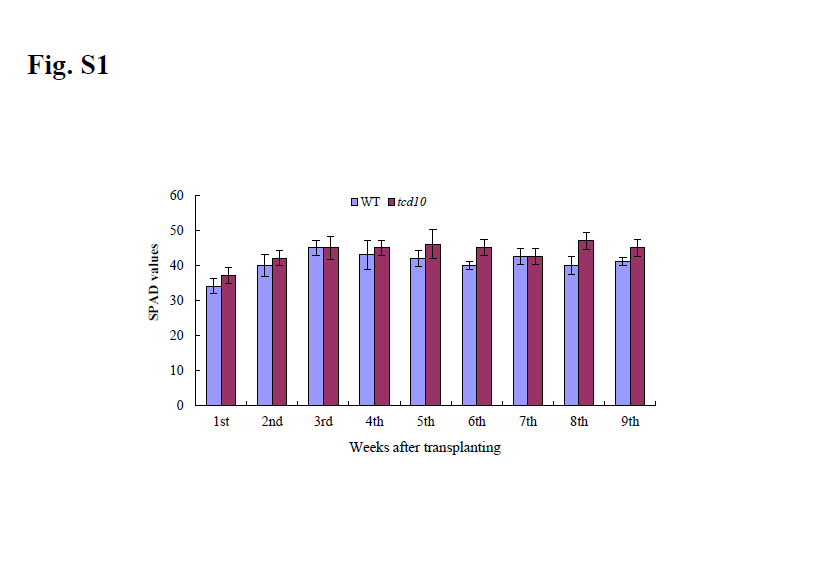

Supplement: Additional file 1: — Figure S1. Changes of leaf chlorophyll SPAD from transplanting to maturity. (2010, Shanghai, China). (DOC 43 kb) [file 12284_2016_134_MOESM1_ESM.doc]

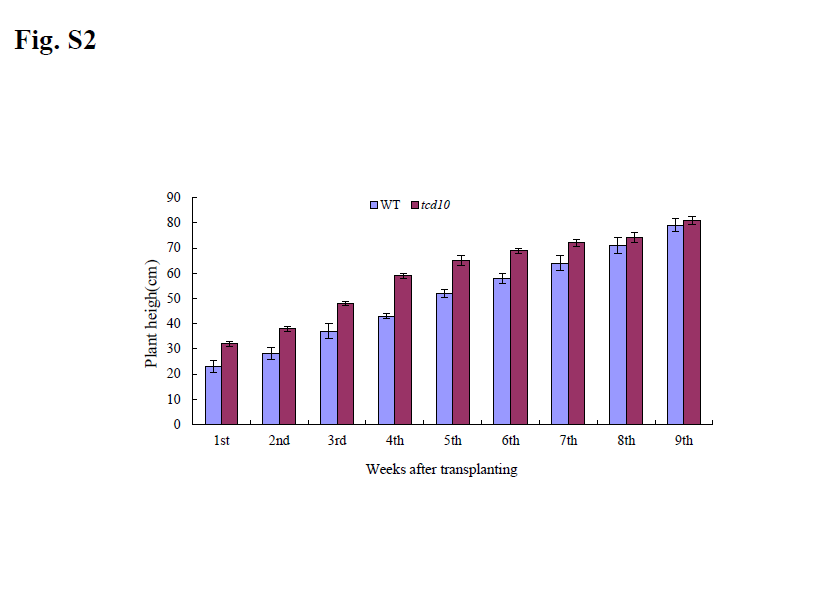

Supplement: Additional file 2: — Figure S2. Changes of plant height from transplanting to maturity. (2010, Shanghai, China). (DOC 47 kb) [file 12284_2016_134_MOESM2_ESM.doc]

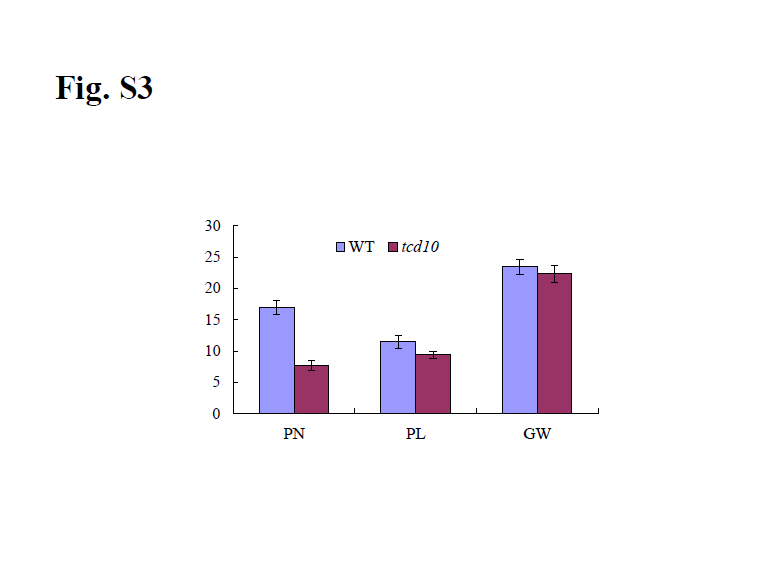

Supplement: Additional file 3: — Figure S3. Comparison of panicle-related traits between tcd10 mutant and wild type (2010, Shanghai, China); PN, Panicle number; PL, Panicle length (cm); GW, 1000-grain weight (g). (DOC 38 kb) [file 12284_2016_134_MOESM3_ESM.doc]

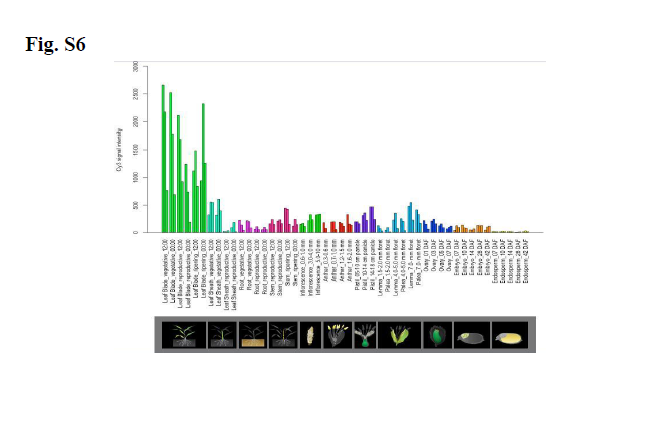

Supplement: Additional file 8: — Figure S6. Expression patterns of TCD10 (LOC_Os10g28600). Data were obtained from the rice expression profile database, RiceXPro (http://ricexpro.dna.affrc.go.jp/category-select.php). (DOC 98 kb) [file 12284_2016_134_MOESM8_ESM.doc]
