# Supplementary material for: The Rice Pentatricopeptide Repeat Gene TCD10 is Needed for Chloroplast Development under Cold Stress
Source: Rice (N Y). 2016 Dec 1;9:67. doi: 10.1186/s12284-016-0134-1 (PMC5133210; doi:10.1186/s12284-016-0134-1)
Supplement: Additional file 4: — Table S1. Genetic segregation of tcd10 mutants in the F2 population. (DOC 30 kb) [file 12284_2016_134_MOESM4_ESM.doc]

**Table S1**. Genetic segregation analysis of *tcd10* mutants in the F_2_ population.

| Cross | Observed number of F_2_ plants | | | χ2_(3:1)_ |
| --- | --- | --- | --- | --- |
|  | Total | Green | Albino |  |
| Guangzhen63/*tcd10* | 3196 | 2376 | 820 | 0.80 |

χ2_0.05_=3.84
