# Supplementary material for: The Rice Pentatricopeptide Repeat Gene TCD10 is Needed for Chloroplast Development under Cold Stress
Source: Rice (N Y). 2016 Dec 1;9:67. doi: 10.1186/s12284-016-0134-1 (PMC5133210; doi:10.1186/s12284-016-0134-1)
Supplement: Additional file 5: — Table S2. The PCR-based molecular markers designed for fine mapping. (DOCX 14 kb) [file 12284_2016_134_MOESM5_ESM.docx]

**Table S2** The PCR-based molecular markers designed for fine mapping

| Marker | Name | Primer sequences |
| --- | --- | --- |
| SSR | RM311 | 5'-CCAGGACACACTGTGAGTAGTTTGC-3' |
|  |  | 5'-TTTCCCGTATGACAACACACTGC-3' |
|  | RM25441 | 5'-GTTAGGTTCAGGACGAGGGC-3' |
|  |  | 5'-CGAAGCCCACCACAATATCT-3' |
|  | RM333 | 5'-GTAGGCTTGTCACCTTCCCTTCC-3' |
|  |  | 5'-GGTCCTGCAGAAAGAATCAAAC-3' |
|  | RM209 | 5'-GGTGATTAATTACTGGTCGGAAGG-3' |
|  |  | 5'-GCTGGTTTGATCGGAATTACAGG-3' |
| InDel | ID14765 | 5'-CAGAACAACTGGTCTAAATGATGCAA-3' |
|  |  | 5'-CGCGATTACATATGTCCCAGTT-3' |
|  | ID14800 | 5'-TGATTCCTCGACGAAGGCAGGTACA-3' |
|  |  | 5'-GCCGTCCAGATCATGCATGAGAAAA-3' |
|  | ID14874 | 5'-AGATCGATCGAATGGACAGC-3' |
|  |  | 5'-GGAATGGGAGTAGTTGGGGT-3' |
|  | ID14944 | 5'-TGAGGCAGAGACAAGCAGGGGCA-3' |
|  |  | 5'-CATCTTCCTCCCCAATAACAAAATC-3' |
|  | ID15439 | 5'-GGTACATTGCAGTGCGGAAGC-3' |
|  |  | 5'-ATCTTCGAACACGCGAACACG-3' |
