# Supplementary material for: The Rice Pentatricopeptide Repeat Gene TCD10 is Needed for Chloroplast Development under Cold Stress
Source: Rice (N Y). 2016 Dec 1;9:67. doi: 10.1186/s12284-016-0134-1 (PMC5133210; doi:10.1186/s12284-016-0134-1)
Supplement: Additional file 6: — Figure S4. Full-length cDNA sequence of TCD10 and mutation sites; The nucleotides with the blue (A), red (G) and green (CCATGGCCGGGTCGGGG) letters represent the deleted nucleotides in tcd10, T1-1 and T1-2 transgenic lines using CRISPR/Cas9 system technique; The sequences in the box represents the recognition sequences in CRIPPER/Cas9 experiments. (DOCX 21 kb) [file 12284_2016_134_MOESM6_ESM.docx]

Fig. S4 Full-length cDNA sequence of *TCD10* and mutation sites

1 ATGTTGGAGGTCTGCTGCTGCTCCGGCGTCCTCGGCGGGTCGCCGCCGTCGTCGAGGACT

61 GCGGGGGTTTCCTCACCTGGACTATCGCCGTCTCGGCCCAGTAAGCGGCGAATCGGCCGC

121 GCCCGGGTGCAGCCGCGCGCGCCGCCGCCGTGCGACGAGCGGAGGGCGGCGGAGGACGTC

181 ATCCACGCGCTCAGGTCGGCGGATGGCCCCGCCGAGGCGCTTGAGCGGTTCAGGTCGGCG

241 GCGCGGAAGCCCAGGGTGGCCCACACGACCGCGTCGTGCAACTACATGCTCGAGCTCATG

301 CGCGGCCATGGCCGGGTCGGGGACATGGCCGAGGTGTTCGACGTAATGCAGAGGCAGATC

361 GTCAAGGCGAACGTGGGCACGTTCGCGGCGATCTTCGGAGGGCTTGGTGTGGAGGGGGGA

421 CTCCGGAGCGCGCCGGTGGCGCTGCCGGTCATGAAGGAGGCCGGGATTGTCTTGAACGCG

481 TACACGTATAATGGCCTGGTTTATTTCCTTGTGAAGTCCGGGTTTGATAGGGAGGCGTTG

541 GAGGTTTATAGGGTGATGATGGTGGATGGTGTTGTGCCTAGTGTGAGAACCTACTCTGTG

601 CTGATGGTGGCGTTCGGGAAGAGGAGGGATGTCGAGACGGTTCTTTGGTTGTTGCGTGAA

661 ATGGAGGCTCATGGCGTGAAGCCGAATGTGTATAGCTACACCATCTGTATTCGAGTTCTT

721 GGACAAGCCAAAAGATTCGATGAAGCTTATCGGATACTTGCGAAA**A**TGGAGAATGAAGGG

781 TGTAAGCCGGATGTCATTACCCATACCGTGCTTATACAGGTTCTTTGCGATGCTGGCCGT

841 ATCAGTGATGCCAAGGATGTTTTTTGGAAGATGAAGAAGAGTGATCAAAAACCTGATCGA

901 GTGACCTACATTACTCTGTTAGATAAGTTTGGTGACAATGGTGACTCGCAATCAGTGATG

961 GAAATCTGGAATGCAATGAAAGCTGATGGGTACAATGACAATGTTGTTGCTTATACAGCA

1021 GTTATTGATGCATTGTGCCAAGTTGGGAGGGTCTTTGAAGCTTTGGAAATGTTTGATGAG

1081 ATGAAACAAAAGGGTATAGTGCCTGAGCAGTATTCATACAACTCGCTGATATCGGGGTTT

1141 CTTAAAGCTGATAGATTTGGTGATGCCTTAGAGCTGTTTAAACATATGGATATTCATGGA

1201 CCTAAACCAAATGGTTACACACATGTTCTTTTCATAAATTACTATGGAAAATCTGGTGAA

1261 TCTATAAAGGCAATACAGAGATATGAACTAATGAAGAGCAAAGGGATTGTGCCAGATGTT

1321 GTTGCTGGTAATGCTGTTTTGTTTGGTCTTGCCAAATCTGGTAGACTTGGCATGGCAAAA

1381 AGGGTATTTCATGAATTAAAAGCTATGGGGGTTTCTCCGGATACTATCACCTACACTATG

1441 ATGATCAAGTGTTGCAGCAAGGCATCAAAATTTGATGAAGCTGTGAAGATTTTCTATGAT

1501 ATGATTGAAAATAATTGTGTTCCTGATGTTCTTGCCGTGAATTCTTTGATTGATACACTC

1561 TACAAGGCAGGCAGGGGTGATGAAGCCTGGCGGATCTTTTATCAACTAAAAGAAATGAAT

1621 CTAGAGCCAACAGATGGGACTTACAATACACTTTTGGCAGGATTGGGAAGGGAAGGTAAA

1681 GTCAAGGAGGTAATGCATCTGCTTGAAGAAATGTACCACAGTAATTATCCTCCTAATTTG

1741 ATAACATACAATACGATTCTTGACTGTCTCTGCAAGAACGGGGCAGTTAATGATGCACTT

1801 GATATGCTATACAGTATGACTACGAAAGGATGCATACCTGATCTTTCGTCTTACAACACT

1861 GTTATATATGGCCTTGTTAAAGAAGAAAGATATAATGAGGCATTCAGTATTTTTTGTCAG

1921 ATGAAGAAGGTTCTTATTCCAGATTATGCAACACTGTGTACTATCCTCCCAAGTTTTGTG

1981 AAAATTGGACTGATGAAGGAAGCTCTGCATATTATCAAGGACTACTTCCTCCAACCTGGC

2041 TCCAAAACAGATAGGTCTTCATGCCATTCACTAATGGAAGGGATACTGAAGAAGGCTGGC

2101 ATCGAAAAGTCAATTGAGTTTGCTGAAATCATAGCATCAAGTGGTATTACCTTGGATGAT

2161 TTCTTTTTGTGCCCATTAATTAAGCATCTCTGTAAGCAGAAGAAAGCTCTTGAAGCACAT

2221 GAACTTGTCAAAAAGTTCAAGAGCTTTGGAGTTTCACTAAAAACTGGATTATATAATTCT

2281 TTGATTTGTGGGCTTGTTGATGAAAACCTAATAGATATTGCTGAAGGCTTGTTTGCTGAA

2341 ATGAAGGAACTCGGTTGTGGTCCAGATGAGTTTACTTACAACTTACTTCTTGATGCCATG

2401 GGAAAGTCAATGCGGATAGAGGAAATGCTAAAAGTTCAAGAAGAGATGCATCGCAAGGGA

2461 TATGAATCAACTTATGTTACTTATAACACAATCATTTCAGGTCTTGTAAAGTCAAGAAGG

2521 TTGGAGCAGGCTATTGATTTGTACTACAACCTGATGAGCCAAGGTTTCTCACCCACACCA

2581 TGCACATATGGCCCTCTTCTTGATGGTCTGTTAAAAGCTGGAAGGATAGAAGATGCAGAA

2641 AATCTTTTCAATGAGATGCTGGAGTATGGATGCAAGGCCAATTGCACTATCTACAATATA

2701 CTACTGAATGGACATCGAATAGCTGGTAATACAGAGAAGGTCTGTCATTTGTTTCAGGAT

2761 ATGGTTGACCAGGGAATAAACCCAGATATAAAATCCTACACGATTATTATTGACACACTC

2821 TGCAAGGCAGGACAGTTAAATGATGGTCTAACATATTTTAGGCAATTATTAGAAATGGGT

2881 CTTGAACCTGATCTAATTACTTACAATTTGCTCATTGATGGTCTTGGAAAATCAAAAAGA

2941 TTAGAGGAAGCAGTGTCTCTATTCAATGAGATGCAGAAGAAGGGAATTGTCCCGAACTTG

3001 TACACTTATAATTCACTAATTCTCCACTTAGGAAAAGCAGGGAAGGCTGCTGAAGCTGGG

3061 AAAATGTATGAAGAGCTACTGACGAAAGGCTGGAAGCCTAACGTTTTCACATATAACGCT

3121 CTTATTAGGGGGTACAGTGTTTCCGGCAGTACTGATAGTGCCTATGCTGCCTATGGTCGG

3181 ATGATTGTTGGCGGGTGCCTACCCAATTCAAGCACGTACATGCAGCTCCCAAATCAGCTG

3241 TGA
