# Supplementary material for: The Rice Pentatricopeptide Repeat Gene TCD10 is Needed for Chloroplast Development under Cold Stress
Source: Rice (N Y). 2016 Dec 1;9:67. doi: 10.1186/s12284-016-0134-1 (PMC5133210; doi:10.1186/s12284-016-0134-1)
Supplement: Additional file 7: — Figure S5. The protein sequences of TCD10 and PGR3 (At4g31850); The sequences with green letters indicate chloroplast transit peptide (CTP); The numbers represent PPR motifs; In TCD10, the amino acid with red letter indicate the deletion mutation sites in tcd10 (the 1st PPR) and T1-1, T1-2 transgenic lines (the 5th PPR), respectively; In PGR3, the amino acid with red letter indicate the substitute mutation sites in pgr3-1(the 15th PPR), pgr3-2(the 12th PPR) and pgr3-3(the 27th PPR) mutants. (DOCX 21 kb) [file 12284_2016_134_MOESM7_ESM.docx]

Fig. S5 The sequences of TCD10 and PGR3

TCD10(LOC_Os10g28600) MLEVCCCSGVLGGSPPSSRTAGVSSPGLSPSRPSKRRIGRARVQPRAPPPCDERRAAEDVIHALRSADGPAEALERFRSAARKPRVAHTTASCNYMLELMRGHGRVGDMAEVFDVMQRQIV^1^KANVGTFAAIFGGLGVEGGLRSAPVALPVMKEAGI^2^VLNAYTYNGLVYFLVKSGFDREALEVYRVMMVDGV^3^VPSVRTYSVLMVAFGKRRDVETVLWLLREMEAHGV^4^KPNVYSYTICIRVLGQAKRFDEAYRILAKMENEGC^5^KPDVITHTVLIQVLCDAGRISDAKDVFWKMKKSDQ^6^KPDRVTYITLLDKFGDNGDSQSVMEIWNAMKADGY^7^NDNVVAYTAVIDALCQVGRVFEALEMFDEMKQKGI^8^VPEQYSYNSLISGFLKADRFGDALELFKHMDIHGP^9^KPNGYTHVLFINYYGKSGESIKAIQRYELMKSKGI^10^VPDVVAGNAVLFGLAKSGRLGMAKRVFHELKAMGV^11^SPDTITYTMMIKCCSKASKFDEAVKIFYDMIENNC^12^VPDVLAVNSLIDTLYKAGRGDEAWRIFYQLKEMNL^13^EPTDGTYNTLLAGLGREGKVKEVMHLLEEMYHSNY^14^PPNLITYNTILDCLCKNGAVNDALDMLYSMTTKGC^15^IPDLSSYNTVIYGLVKEERYNEAFSIFCQMKKVLI^16^PDYATLCTILPSFVKIGLMKEALHIIKDYFLQPGSKTDRSSCHSLMEGILKKAGIEKSIEFAEIIASSGI^17^TLDDFFLCPLIKHLCKQKKALEAHELVKKFKSFGVSLKTGLYNSLICGLVDENLIDIAEGLFAEMKELGC^18^GPDEFTYNLLLDAMGKSMRIEEMLKVQEEMHRKGY^19^ESTYVTYNTIISGLVKSRRLEQAIDLYYNLMSQGF^20^SPTPCTYGPLLDGLLKAGRIEDAENLFNEMLEYGC^21^KANCTIYNILLNGHRIAGNTEKVCHLFQDMVDQGI^22^NPDIKSYTIIIDTLCKAGQLNDGLTYFRQLLEMGL^23^EPDLITYNLLIDGLGKSKRLEEAVSLFNEMQKKGI^24^VPNLYTYNSLILHLGKAGKAAEAGKMYEELLTKGW^25^KPNVFTYNALIRGYSVSGSTDSAYAAYGRMIVGGC^26^LPNSSTYMQLPNQL^27^

PGR3 (At4g31850)

MVALLCSASLCGDISVGDACLVSNIKAKCRDNLVTGGLKFHALKIGSRKKHWRRKSMRCSVVSMKSSDFSGSMIRKSSKPDLSSSEEVTRGLKSFPDTDSSFSYFKSVAGNLNLVHTTETCNYMLEALRVDGKLEEMAYVFDLMQKRII**^1^**KRDTNTYLTIFKSLSVKGGLKQAPYALRKMREFGF^2^VLNAYSYNGLIHLLLKSRFCTEAMEVYRRMILEGF^3^RPSLQTYSSLMVGLGKRRDIDSVMGLLKEMETLGL^4^KPNVYTFTICIRVLGRAGKINEAYEILKRMDDEGC^5^GPDVVTYTVLIDALCTARKLDCAKEVFEKMKTGRH^6^KPDRVTYITLLDRFSDNRDLDSVKQFWSEMEKDGH^7^VPDVVTFTILVDALCKAGNFGEAFDTLDVMRDQGI^8^LPNLHTYNTLICGLLRVHRLDDALELFGNMESLGV^9^KPTAYTYIVFIDYYGKSGDSVSALETFEKMKTKGI^10^APNIVACNASLYSLAKAGRDREAKQIFYGLKDIGL^11^VPDSVTYNMMMKCYSKVGEIDEAIKLLSEMMENGC^12^EPDVIVVNSLINTLYKADRVDEAWKMFMRMKEMKL^13^KPTVVTYNTLLAGLGKNGKIQEAIELFEGMVQKGC^14^PPNTITFNTLFDCLCKNDEVTLALKMLFKMMDMGC^15^VPDVFTYNTIIFGLVKNGQVKEAMCFFHQMKKLVY^16^PDFVTLCTLLPGVVKASLIEDAYKIITNFLYNCADQPANLFWEDLIGSILAEAGIDNAVSFSERLVANGI^17^CRDGDSILVPIIRYSCKHNNVSGARTLFEKFTKDLGVQPKLPTYNLLIGGLLEADMIEIAQDVFLQVKSTGC^18^IPDVATYNFLLDAYGKSGKIDELFELYKEMSTHEC^19^EANTITHNIVISGLVKAGNVDDALDLYYDLMSDRD^20^FSPTACTYGPLIDGLSKSGRLYEAKQLFEGMLDYGC^21^RPNCAIYNILINGFGKAGEADAACALFKRMVKEGV^22^RPDLKTYSVLVDCLCMVGRVDEGLHYFKELKESGL^23^NPDVVCYNLIINGLGKSHRLEEALVLFNEMKTSRG^24^ITPDLYTYNSLILNLGIAGMVEEAGKIYNEIQRAGL^25^EPNVFTFNALIRGYSLSGKPEHAYAVYQTMVTGGF^26^SPNTGTYEQLPNRA^27^
