# Supplementary material for: The Rice Pentatricopeptide Repeat Gene TCD10 is Needed for Chloroplast Development under Cold Stress
Source: Rice (N Y). 2016 Dec 1;9:67. doi: 10.1186/s12284-016-0134-1 (PMC5133210; doi:10.1186/s12284-016-0134-1)
Supplement: Additional file 9: — Table S3. Markers designed for realtime RT-PCR and gene functions. (DOCX 18 kb) [file 12284_2016_134_MOESM9_ESM.docx]

**Table S3** Markers designed for realtime RT-PCR and gene function

| Gene | Primer(5’-3’) | Gene function |
| --- | --- | --- |
| *TCD10* | TCGGTTGTGGTCCAGATGAGT | Encoding a novel pentatricopeptide repeat protein  in his study |
|  | ATCTGGGTTTATTCCCTGGTC |  |
| *Cab1R* | AGATGGGTTTAGTGCGACGAG | Encoding light-harvesting chlorophyll alb binding protein 1 (Luan and Bogorad, 1989) |
|  | TTTGGGATCGAGGGAGTATTT |  |
| *Cab2R* | TGTTCTCCATGTTCGGCTTCT | Encoding light-harvesting chlorophyll alb binding protein 2 (Luan and Bogorad, 1989) |
|  | GCTACGGTCCCCACTTCACT |  |
| *CAO* | GATCCATACCCGATCGACAT | The chlorophyllide a oxygenase in Chl b biosynthesis (Oster et al.2000) |
|  | CGAGAGACATCCGGTAGAGC |  |
| *FtsZ* | AAAGGACATAACCTTGCAAG | Encoding a component of the plastid division machinery (Takeuchi et al. 2007 ) |
|  | AGTTTTCCTATTGAACCGTG |  |
| *LhcpII* | GAAGAAGATCAAGAACGGCC | Encoding light harvesting chlorophyll a⁄b binding protein of PSII (Murray and Kohorn 1991) |
|  | TTGCCGGGGACGAAGTTGGT |  |
| *OsDG2* | AAACCGAAATCGTCGTGGAG | Encoding a novel chloroplast-targeted GR protein  (Jiang et al.2014) |
|  | ACAAGGGAGCACCTGAACTAAGA |  |
| *OsRpoLP1* | ACCGGTGCTTTCAGGCTTGG | Encoding one plastidial DNApolymerase  (Takeuchi et al. 2007) |
|  | GCTGACTGATAATCACACG |  |
| *OsRpoTp* | AAGCAGACAGTGATGACATC | Encoding RNA polymerase subunit of PEP (Hiratsuka et al. 1989) |
|  | ATCACATGCATGCACCCAAA |  |
| *OsV4* | ACATGGTCGCCGTCTTCCGC | Encoding a novel pentatricopeptide repeat protein  (Gong et al.2014). |
|  | GCTTGCCAGCACTGTCACGA |  |
| *PORA* | TGTACTGGAGCTGGAACAACA | Encoding pchlide oxidoreductase (POR)  (Sperling, et al.1997) |
|  | GAGCACAGCAAAATCCTAGACG |  |
| *psaA* | GCGAGCAAATAAAACACCTTC | Encoding the P700 apoproteins of photosystem I (Steiner et al. 2009) |
|  | GTACCAGCTTAACGTGGGGAG |  |
| *psbA* | CCCTCATTAGCAGATTCGTTTT | Encoding the D1 protein of photosystem II (Steiner et al. 2009) |
|  | ATGATTGTATTCCAGGCAGAGC |  |
| *rbcL* | CTTGGCAGCATTCCGAGTAA | Encoding the large subunit of Rubisco  (Barkan et al.1993) |
|  | ACAACGGGCTCGATGTGATA |  |
| *rbcS* | TCCGCTGAGTTTTGGCTATTT | Encoding ribulose-bisphosphate carboxylase  (Wanner et al.1991) |
|  | GGACTTGAGCCCTGGAAGG |  |
| *rpl21(ASL2)* | AAGAAGAGGAGGCTGCGGT | Encoding the plastid ribosomal protein L21  ( Lagrange et al.1997; Lin et al., 2015) |
|  | GACATTGGCGCCTTTCAGC |  |
| *rpoB* | TTTGGTTTCGATGTGCA | Encoding RNA polymerase β subunits of PEP  (Little et al. 1988) |
|  | TATGGTCTAATTCCGAGCGGT |  |
| *rpoC* | ATTAGACGCATGCAATTGGC | Encoding RNA polymerase β' subunits of PEP  (Little et al. 1988) |
|  | CAATGGGTCTTAATTCGGGA |  |
| *rps7* | GCCAAAATCCATTCCAATTC | Encoding Chloroplast-encoded small ribosomal protein subunit 7 |
|  | GGAGATGTACACGAGGAGATTG |  |
| *rps20(ASl1)* | CACGCTCTTCTCCCTCTCCT | Encoding a component (S20) of the small ribosomal subunit (Nieminen et al .2011, Gong et al. 2013) |
|  | GTAGGAGGCGGACAGGCG |  |
| *RNRL* | GTTAGATGCTTCACTACACAG | Gene encodes the large subunit of ribonucleotide reductase (V3) (Yoo et al. 2009) |
|  | GTACCATTGCCAACATGGCAAC |  |
| *RNRS* | GCCAAAATCCATTCCAATTC | Enconding the small subunit of ribonucleotide reductase 1*(st1*) (Yoo et al.2009) |
|  | GGAGATGTACACGAGGAGATTG |  |
| *V1* | TAGTGGCTGTTGCCAGTGGA | Encoding a chloroplast localized protein NUS1  (Kusumi et al.1997) |
|  | TATCTAGTCTAACCACCAGC |  |
| *V2* | GAGGAGTTCCTCACGATGAT | Encoding a new type of plastid/mitochondrial guanylate kinase (pt/mt GK) (Sugimoto et al. 2007) |
|  | AGCATCAATGATAGACTCC |  |
| *YGL1* | CAGTCTCCAATGGCCACCT | Encoding chlorophyll synthesis(Wu et al.2007) |
|  | TGCTTTCATCAGTGGCTGGT |  |
| *16sRNA* | CCGTTGGTGTTCTTTCCGAT | Encoding chloroplast ribosomes small subunits of 16S components |
|  | TTCAAGTCCGCCGTCAAATC |  |
| *23sRNA* | TGTGGGCGTTAGAGCATTGAG | Encoding chloroplast ribosomes large subunits of 23Scomponents |
|  | CACTTGGCTACCCAGCGTTTA |  |
| *OsActin* | AGGAAGGCTGGAAGAGGACC | Encoding chlorophyll synthesis (Wu et al.2007) |
|  | CGGGAAATTGTGAGGGACAT |  |
